# From Sensory Regulation to Participation: A Clinical Reasoning Framework for Aquatic Therapy in Children with Autism Spectrum Disorder

**DOI:** 10.3390/children13101327

**Published:** 2026-09-30

**Authors:** Javier Güeita-Rodríguez, Anna Ogonowska-Slodownik

**Affiliations:** 1 Research Group of Humanities and Qualitative Research in Health Science (Hum&QRinHS), Department of Physiotherapy, Occupational Therapy, Rehabilitation, and Physical Medicine, Universidad Rey Juan Carlos, 28922 Madrid, Spain; 2 Faculty of Rehabilitation, Jozef Pilsudski University of Physical Education in Warsaw, 00-968 Warsaw, Poland; anna.ogonowskaslodownik@awf.edu.pl; 3 KITE Research Institute, Toronto Rehabilitation Institute, University Health Network, Toronto, ON M5G 2A2, Canada

**Keywords:** autism spectrum disorder, aquatic therapy, clinical reasoning, evidence-based practice, narrative review, outcome measures

## Abstract

**Highlights:**

**What are the main findings?**

- Current evidence indicates that aquatic interventions may address multiple domains in children with autism spectrum disorder (ASD), including sensory regulation, motor performance, aquatic competence, social interaction, and participation.
- This review synthesizes the available evidence into an evidence-informed five-phase clinical reasoning framework linking therapeutic priorities, aquatic activities, and meaningful outcomes.

**What are the implications of the main findings?**

- The proposed framework may help clinicians translate heterogeneous evidence into individualized aquatic therapy planning based on children’s needs, goals, and participation.
- A shared clinical reasoning framework may improve consistency in aquatic therapy practice and provide a conceptual basis for future research on intervention components, outcomes, and mechanisms in children with ASD.

**Abstract:**

Background and Objective: Research on aquatic interventions for children with autism spectrum disorder (ASD) has spanned over four decades, yet the field remains characterized by a fragmented evidence where therapeutic techniques often coexist without a unifying conceptual logic. This has led to a translational gap between research findings and clinical decision-making. This narrative review aimed to synthesize four decades of research into an evidence-informed clinical reasoning framework to capture the evolution of aquatic therapeutic practice and support clinical decision-making. Methods: Literature search was conducted in PubMed, Scopus, and Web of Science, covering publications from the earliest available records to May 2026. Studies were analysed according to their therapeutic concepts, clinical reasoning logic, and outcome domains. Results: A total of 30 studies met the inclusion criteria and were included in this review. The resulting synthesis enabled the derivation of an evidence-informed clinical reasoning framework representing five dominant therapeutic perspectives that characterize the development of aquatic clinical reasoning across the literature. By synthesizing peer-reviewed literature, we identified five dominant clinical reasoning paradigms that emerged across the development of the literature and continue to inform contemporary aquatic practice: (1) Exploratory and Sensory-Regulatory, (2) Skill- and Safety-Oriented, (3) Structured and Functional, (4) Participation- and Social-Oriented, and (5) Individualized and Outcome-Informed Practice. Conclusions: This conceptual roadmap may help clinicians align aquatic intervention strategies with individual therapeutic priorities, functional goals, and participation needs. The proposed framework provides a bridge between historical practice and contemporary evidence, supporting individualized and outcome-informed clinical reasoning in aquatic therapy for children with ASD. The five phases should be interpreted as dominant paradigms of clinical reasoning that may coexist within practice rather than as mutually exclusive or strictly chronological categories.

## 1. Introduction

Autism Spectrum Disorder (ASD) is a neurodevelopmental condition characterized by persistent differences in social communication and social interaction, together with restricted, repetitive patterns of behaviour, interests, or activities [1]. In addition, many autistic children experience sensory processing differences that may influence participation in daily activities, leisure occupations, educational contexts, and community environments. These challenges contribute to the wide heterogeneity observed across individuals on the autism spectrum and frequently affect engagement in meaningful activities and social participation. Hume et al. (2021) highlighted that participation outcomes remain a central priority for intervention and support across the lifespan [2]. Although the presentation of ASD is highly heterogeneous, challenges related to participation often emerge from the dynamic interaction between individual characteristics, contextual demands, and environmental factors. These considerations are particularly relevant in aquatic contexts, where the sensory, motor, social, and environmental properties of water may create unique opportunities to support engagement, skill development, safety, and meaningful participation [3].

The aquatic environment, characterized by its unique physical properties (including buoyancy, hydrostatic pressure, viscosity, and thermal conductivity) provides a rich multisensory context that differs substantially from land-based environments [4]. These characteristics facilitate movement, modulate sensory input, and create opportunities for motor, social, and behavioral engagement that are particularly relevant for individuals with autism spectrum disorder (ASD). However, as the field has matured, there has been a significant shift in how clinicians and researchers conceptualize the “why” and “how” of aquatic therapy. Aquatic interventions for children with ASD have evolved from simple recreational activities into a specialized field of therapeutic practice, characterized by unique sensory, motor, and social affordances. This evolution is not an accumulation of techniques, but a transformation in clinical reasoning: the cognitive process through which clinicians align therapeutic goals with specific outcome variables.

Existing systematic reviews, such as those by Mortimer et al. [5] and Van ’t Hooft et al. [3], have provided critical evidence-based aggregations of intervention efficacy and effect sizes. However, they primarily focused on what outcomes are achieved rather than the underlying how and why of the therapeutic process. Our narrative review complements these systematic efforts by addressing the current void in clinical reasoning, offering a conceptual roadmap that explains the evolutionary logic behind goal selection and the mapping of variables, thus bridging the gap between statistical evidence and clinical decision-making. Accordingly, our contribution is conceptual and translational, clarifying why certain goals and outcomes have been prioritized over time and how those priorities can inform present-day clinical decision-making in aquatic contexts for ASD. Recent review further reflect this contemporary perspective by describing aquatic intervention as a multimodal and individualized approach that simultaneously targets sensory regulation, motor competence, social participation, and wellbeing while emphasizing the need for standardized protocols, therapist training, and meaningful outcome monitoring [3].

Despite the increasing number of intervention studies, translating research findings into clinical decision-making remains challenging [2]. Existing aquatic programs differ substantially in their therapeutic objectives, intervention components, dosage, outcome measures, and theoretical rationale, making it difficult for clinicians to identify which approaches are most appropriate for specific clinical goals [3]. This review responds to this need by offering a clinically oriented interpretation of the literature and an evidence-informed framework intended to facilitate knowledge translation and clinical decision-making. Consequently, clinicians lack a coherent framework to guide intervention planning and translate research into practice. Therefore, the aim of this narrative review was to synthesize research and derive an evidence-informed clinical reasoning framework organized around three complementary dimensions (therapeutic concepts, clinical decision-making logic, and outcome mapping) that captures the evolution of aquatic therapeutic practice and supports clinical decision-making, knowledge translation, and future research.

## 2. Materials and Methods

This study employed a narrative review using a structured narrative synthesis approach to examine the evolution of aquatic interventions for children with ASD. This approach was selected because it enables the integration of conceptually diverse literature into a coherent clinical framework while preserving the historical development of therapeutic concepts, clinical decision-making, and outcome priorities. The review was developed in accordance with the Scale for the Assessment of Narrative Review Articles (SANRA) to enhance methodological transparency, reporting quality, and interpretative rigor [6].

### 2.1. Search Strategy

A narrative review with a structured narrative synthesis approach was undertaken to examine the evolution of aquatic interventions for children with ASD. The objective was not to comprehensively identify all available studies, but rather to synthesize the conceptual development of aquatic therapeutic approaches over time and derive an evidence-informed clinical reasoning framework.

A targeted literature search was conducted in PubMed, Scopus, and Web of Science, covering publications from the earliest available records to May 2026. Search terms combined concepts related to autism spectrum disorder, aquatic therapy, hydrotherapy, aquatic exercise, swimming, water-based intervention, pediatric rehabilitation, and clinical reasoning. Reference lists of relevant publications were also manually screened to identify additional studies of conceptual relevance.

### 2.2. Eligibility Criteria

Studies were considered eligible if they: (a) investigated aquatic interventions involving children or adolescents diagnosed with ASD; (b) described therapeutic objectives, intervention characteristics, or clinical outcomes; (c) contributed conceptually to the evolution of aquatic therapeutic practice. Conceptual relevance was defined as the study’s contribution to understanding therapeutic rationale, intervention priorities, clinical decision-making processes, or outcome domains relevant to the development of aquatic clinical reasoning in ASD.

Given the conceptual purpose of the review, no restrictions were imposed regarding study design. Randomized controlled trials, non-randomized studies, observational studies, pilot studies, case series, and clinically relevant descriptive reports were considered when they provided meaningful information regarding therapeutic concepts or intervention strategies.

Studies focusing exclusively on competitive swimming or non-therapeutic recreational activities were excluded.

### 2.3. Data Extraction and Narrative Synthesis

For each included study, information was extracted regarding therapeutic rationale, intervention objectives, characteristics of the aquatic intervention, outcome domains evaluated and implications for clinical decision-making.

Rather than quantitatively comparing intervention effectiveness, the included studies were examined using a structured narrative synthesis to identify recurring therapeutic concepts and shifts in clinical priorities across the literature. Particular attention was given to how intervention goals, therapist roles, and outcome measures evolved over successive decades.

A summary of study characteristics, intervention features, outcome domains, and their contribution to the proposed clinical reasoning framework is provided in Supplementary Table S1.

### 2.4. Framework Development

The proposed framework emerged through an iterative process of interpretative and conceptual synthesis aimed at identifying recurring patterns in aquatic intervention research rather than evaluating intervention effectiveness.

Initially, the included studies were organized chronologically to explore how therapeutic priorities evolved across the literature. Subsequently, both authors reviewed the studies with particular attention to four analytical dimensions: (1) therapeutic rationale, (2) intervention priorities, (3) clinical decision-making logic, and (4) outcome domains. Studies were allowed to contribute to more than one conceptual category when relevant, reflecting the multidimensional nature of many aquatic interventions. Both authors independently reviewed the included studies and discussed emerging categories until consensus was reached regarding the final framework structure.

Through repeated comparison and discussion, recurrent patterns were grouped into broader clinical reasoning paradigms representing dominant therapeutic orientations described across the literature. These paradigms were subsequently refined and organized according to three complementary dimensions: Therapeutic Concept, Clinical Decision-Making Logic, and Outcome Mapping.

The resulting framework should be interpreted as an evidence-informed conceptual synthesis intended to support clinical reasoning and knowledge translation. It does not represent a validated intervention model, a prescriptive therapeutic sequence, or a formal classification system.

## 3. Results

**Figure 1:**
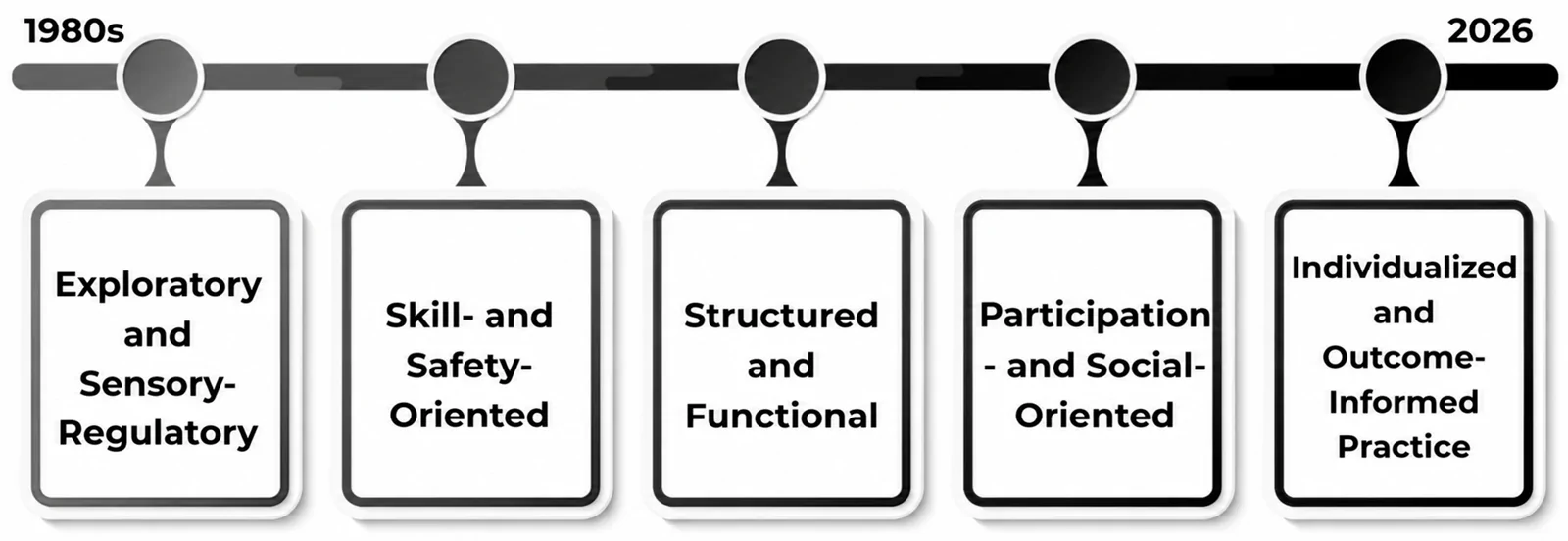
Proposed evidence-informed clinical reasoning framework derived from the reviewed literature.

The structured narrative synthesis identified recurring shifts in therapeutic priorities and clinical reasoning approaches across the aquatic intervention literature for children with ASD. The characteristics of the included studies and their contribution to the proposed framework are presented in Supplementary Table S1. Analysis of therapeutic concepts, clinical decision-making logic, and outcome domains revealed five recurring clinical reasoning paradigms that emerged across the evolution of aquatic intervention research (Figure 1) and constitute the proposed evidence-informed clinical framework (Table 1, Table 2 and Table 3).

### 3.1. Phase 1. Exploratory & Sensory-Regulatory

The initial phase, which we identified as the Exploratory and Sensory-Regulatory Phase, dominated the research landscape from the 1980s through the mid-1990s. During this period, clinical reasoning was primarily focused on the feasibility of the aquatic medium as a sensory-regulatory tool. Seminal work by Killian et al. [7] established the first protocols for measuring water orientation and beginner swim skills. At this phase, the reasoning was “pre-functional,” prioritizing outcomes such as compliance, affective response, and reduction in distress behaviors over mastery of aquatic specific motor techniques.

A closer analysis of intervention descriptions from this period suggests that clinicians were already implicitly leveraging the physical properties of water as regulatory mechanisms. Hydrostatic pressure and viscosity provided graded proprioceptive input, while buoyancy reduced axial loading and postural demand. Warm water immersion was frequently associated with decreased hypertonicity and anxiety, facilitating co-regulation. Although not yet conceptualized in neurobiological terms, these mechanisms supported calm alertness and tolerance of aquatic input.

Interventions were typically delivered in 1:1 formats, often with the same clinician to enhance predictability. Sessions tended to follow short regulatory loops: entry, brief exposure task, preferred activity, structured exit, even if not formally described as such. Weekly exposure appeared to be associated with reductions in internalizing and attentional dysregulation in some reports, providing a foothold for subsequent instructional work. Outcome indicators extended beyond simple participation and included time engaged in water, reduction of avoidance behaviors, and stabilization of behavioral state.

The findings associated with this phase highlight the importance of establishing a foundation of comfort, predictability, and sensory regulation before emphasizing skill acquisition. Contemporary practice suggests that reducing performance demands and creating consistent entry routines may facilitate engagement for children who experience sensory sensitivities or uncertainty in aquatic environments. Clinicians may benefit from monitoring individual signs of sensory overload and adjusting environmental demands accordingly, recognizing that effective participation often depends on achieving an optimal state of regulation rather than immediately targeting specific aquatic skills.

### 3.2. Phase 2. Skill- and Safety-Oriented

As the field transitioned into the Skill- and Safety-Oriented Phase, the logic shifted toward intentional acquisition of aquatic competencies. Reasoning during this period was grounded in the belief that physical fitness and water safety were central to autonomy. Studies by Yılmaz et al. [8,22] and Huettig and Darden-Melton [9] provided empirical evidence that reported improvements in water adjustment and physical fitness when provided with structured instruction. Pan [23] further explored links between physical fitness, sensory processing, and swim skill acquisition. Instructional strategies such as constant time delay procedures strengthened methodological rigor in teaching foundational aquatic skills [24]. Programs increasingly incorporated task analysis, repetition, and criterion-based progression, with safety routines embedded within predictable session structures.

Beyond the measurable motor variables, this phase reveals increasing pedagogical approach. Task analysis and repetition were used to create predictable motor plans, capitalizing on the supportive nature of water to allow protected failure without injury risk. Intensive formats (such as short-term daily camps) began to emerge as approaches that may facilitate skill acquisition, suggesting that dosage may influence motor learning processes.

Session structure became more predictable and safety-driven, often including: warm-up, safety routine, focused skill block, reinforced play, and structured exit. Delivery varied between 1:1 and 1:2 (clinician plus peer/buddy), with consistent pairing enhancing modeling and reinforcement. Criterion-based progression (e.g., float duration before glide introduction) reflected maturation in clinical reasoning.

Outcome assessment expanded to include aquatic skills (entries and exits, floating, breath control, wall-supported kicking, submersion tolerance) and some programs reported time out of water as a proxy for dysregulation. The clinical focus was no longer solely on tolerance, but on building foundational competencies that reduced drowning risk and opened pathways to participation.

The evolution of this phase underscores the continued importance of developing basic aquatic competence and water safety skills. Structured teaching approaches, supported by visual cues and predictable routines, may enhance learning opportunities while reducing cognitive and sensory demands. Repeated practice of foundational aquatic skills can contribute not only to safety but also to increased confidence, autonomy, and participation in aquatic environments. These principles remain highly relevant in contemporary intervention planning.

### 3.3. Phase 3. Structured and Functional

The subsequent Structured and Functional Phase introduced structured approaches and behavioral logic within the pool. Clinical reasoning emphasized the aquatic environment as a structured context supporting behavioral regulation. Prupas et al. [25], Vonder Hulls et al. [10] and Yılmaz et al. [11] demonstrated how structured aquatic routines and methods such as the Halliwick Concept could improve postural control and reduce stereotypical behaviors. Yanardağ et al. [12] further highlighted the pedagogical dimension of aquatic instruction, reinforcing the importance of structured cueing. Ennis [26] demonstrated that structured aquatic programs may provide structured contexts that support behavioral regulation, enhancing sensorimotor adaptation and supporting social engagement in children with ASD. Albadry et al. [27] demonstrated that a 12-week aquatic training program produced greater improvements in gross motor development, quality of life, and autism-related outcomes than a comparable land-based exercise program in young autistic children. All these authors reinforced the role of aquatic environments as structured contexts for promoting functional motor performance and supporting broader developmental outcomes through task-oriented practice and repeated skill engagement.

During this period, predictability itself became a therapeutic ingredient. Visual schedules, consistent task sequencing, and explicit prompt hierarchies were implemented to support motor learning under manageable cognitive load. Prompt fading logs and repetition across sessions illustrated growing attention to independence trajectories.

Hybrid formats emerged, embedding 1:1 instructional pairs within group settings. This allowed individualized support while introducing peer presence, blending procedural rigor with emerging social exposure, with eight-week 60-min programs being among the most commonly reported intervention formats.

Tasks evolved into serial routines (linked glides and kicks, relay micro-sequences, partner drills) designed to enhance sequencing and working memory engagement. Reports increasingly emphasized carryover across sessions and instructors, signaling a shift from isolated skill performance toward functional generalization.

This phase suggests a shift toward promoting functional performance through structured learning experiences that support independence and generalization. Intervention strategies may incorporate sequencing activities, task-specific practice, and gradual fading of prompts to encourage active problem solving and self-directed performance. From a clinical perspective, the emphasis extends beyond the acquisition of isolated aquatic skills toward facilitating their transfer to meaningful daily activities and broader functional outcomes.

### 3.4. Phase 4. Participation- and Social-Oriented

Significant paradigm shift led to the Participation- and Social-Oriented Phase with a substantial increase in publications compared to earlier phases. Fragala-Pinkham et al. [28,29] demonstrated benefits of group-based aquatic exercise, while Chu and Pan [30] and Alaniz et al. [13] advanced peer- and sibling-assisted models targeting social interaction and water safety. Oriel et al. [31,32] documented improvements in sleep behaviors and social competence within structured aquatic social programs. Caputo et al. [14] introduced multisystem aquatic therapy models integrating motor, cognitive, and social targets within comprehensive protocols. This evolution was further reflected in discipline-specific approaches such as Aquatic Speech and Language Therapy, where the aquatic environment was intentionally used to facilitate communication and language outcomes rather than aquatic skills alone [15]. Similarly, Zanobini and Solari conceptualized the aquatic environment as a mediator of communication [16]. The pool was no longer merely a therapeutic medium but an environment that reduced social pressure and facilitated engagement.

Structured aquatic games (such as relay tasks, cooperative floating challenges, and role-based sequences) created natural contingencies for initiation, response, joint attention, and turn-taking. Rather than abandoning structure, successful programs embedded social goals within predictable frameworks, protecting regulation through visual cues and first-then contingencies.

Weekly group formats of eight weeks or more were feasible in educational contexts, and some programs incorporated booster sessions to support transitions into community settings. Outcome assessment expanded to direct counts of initiations and reciprocal turns alongside standardized social competence ratings from parents and staff. Clinical reasoning evolved toward participation within structured group routines rather than isolated social behaviors.

The literature associated with this phase emphasizes participation as a central therapeutic objective. Aquatic activities may provide opportunities to foster social engagement through cooperative games, shared tasks, and structured interactions embedded within predictable routines. Involving peers, siblings, or family members can further support meaningful participation and social connectedness. Rather than targeting social behaviors in isolation, contemporary approaches increasingly position communication and interaction within naturally motivating and socially relevant aquatic experiences.

### 3.5. Phase 5. Individualized and Outcome-Informed Practice

The contemporary landscape is defined by increasingly standardized and fidelity-monitored programming. Ansari et al. [17,33] examined balance and sleep-related outcomes, while Mills et al. [34] explored aquatic therapy effects on mental health behaviors. Marzouki et al. [35] and Zhao et al. [18] extended investigation toward physiological and neurobiological markers, including executive function and BDNF modulation. Munn et al. [36] and Kemp et al. [19] contributed structured program evaluations and randomized controlled evidence supporting water competency acquisition. Kemp et al. further expanded this individualized perspective by demonstrating important relationships between sensory processing characteristics and water competency, highlighting the need to tailor aquatic interventions according to individual sensory and functional profiles [20]. More recently, the AquOTic programme provided evidence that manualized occupational therapy-based aquatic interventions can successfully improve caregiver- and therapist-prioritized goals, illustrating the growing emphasis on individualized goal attainment and outcome-informed clinical decision-making [21].

Beyond the expanded outcome domains, this phase reflects methodological rigor. Protocols specify dosage, sequence, and component delivery while allowing individualized adaptation. Fidelity checklists, prompt-fading documentation, and safety adherence monitoring enhance replicability and internal validity.

Importantly, contemporary reasoning acknowledges dynamic phase-shifting within sessions. Clinicians may revert temporarily to sensory-regulatory strategies during dysregulation while maintaining a structured program. Outcome selection is now domain-matched and hypothesis-driven, combining skill checklists, social competence scales, mental-health indices, and, increasingly, physiological correlates.

Contemporary aquatic practice increasingly emphasizes individualized goal setting, transparent reporting of intervention components, and outcome monitoring aligned with child and family priorities. Emerging studies have explored physiological indicators such as sleep quality, stress-related measures, and neurotrophic factors. Within a neurodiversity-affirming framework, these indicators should be viewed as complementary markers of regulation, wellbeing, adaptation, and participation rather than attempts to normalize autistic characteristics. Clinical decision making is increasingly guided by the alignment between therapeutic goals, contextual factors, and meaningful outcomes for the child and family.

Contemporary aquatic intervention increasingly emphasizes individualized goal setting and outcome monitoring that reflects the priorities of children and their families. Clinical decision-making may be strengthened by selecting outcome measures that align with the intended goals of intervention and by integrating information across multiple domains, including participation, wellbeing, self-regulation, and aquatic competence. This approach encourages clinicians to evaluate intervention success not only in terms of skill acquisition but also through meaningful changes in everyday functioning and quality of life.

## 4. Discussion

The five-phase framework presented in this review suggests that aquatic intervention for children with ASD has evolved from early approaches primarily centred on sensory regulation and behavioural adaptation [7] to a broader range of therapeutic priorities that increasingly incorporated aquatic competence, participation, and multidimensional outcome domains [35]. The framework should be interpreted as a conceptual synthesis of evolving clinical reasoning priorities derived from the available literature rather than as an experimentally validated intervention pathway or a developmental sequence through which children, clinicians, or programs are expected to progress.

Multiple therapeutic priorities may coexist within the same intervention or session, depending on the child’s abilities, preferences, environmental demands, and participation goals. This perspective may help explain the substantial heterogeneity observed across the literature and provides a framework for interpreting why apparently different aquatic interventions may target complementary outcomes.

An important finding of this synthesis is that the aquatic environment should not be considered simply as an alternative location for delivering exercise. Its physical properties (including buoyancy, hydrostatic pressure, resistance, temperature, and water movement) create a distinctive sensory and motor context in which children must continuously adapt their postural, motor, behavioural, and social responses. Early studies emphasised sensory tolerance, behavioural regulation, and adaptation to the aquatic environment, whereas more recent research has increasingly addressed aquatic competence [7], motor performance [27,29], social participation [14,34], sleep [33], and physiological outcomes [18]. This evolution is consistent with a broader movement toward multidimensional intervention models in which the potential effects of aquatic activity are considered across interacting functional domains rather than through a single therapeutic mechanism.

Nevertheless, the evidence does not yet support the assumption that improvements in one domain necessarily translate into improvements in participation or other occupational outcomes. For example, studies reporting improvements in motor performance, stereotypic behaviours, emotional regulation, or sleep provide evidence of potentially meaningful changes, but these outcomes are not interchangeable and may reflect different mechanisms of action [33,35]. Ansari et al. reported improvements in sleep quality alongside reductions in inflammatory cytokines following aquatic exercise [33], while Marzouki et al. demonstrated changes in motor and behavioural outcomes following different aquatic training approaches [35]. These findings broaden the potential explanatory framework for aquatic intervention, but they should not yet be interpreted as establishing specific neurobiological mechanisms underlying clinical improvement.

A further issue emerging from the synthesis is the heterogeneity in how aquatic intervention and treatment success are defined. Studies have differed substantially in intervention content, duration, frequency, professional background of the interventionist, therapeutic rationale, and outcome measures. Outcomes have included aquatic skills, motor performance, behavioural characteristics, social competence, sensory responses, sleep, and physiological variables. This heterogeneity makes direct comparison between interventions difficult and limits the extent to which specific therapeutic components can be identified as responsible for observed changes. The development and use of more standardized descriptions of aquatic interventions and aquatic competence measures may therefore represent an important step toward improving comparability across studies.

The individualized and outcome-informed phase should be interpreted primarily as an emerging direction within the literature rather than a fully established model supported by homogeneous evidence. Although recent studies increasingly emphasize individualized goal setting, fidelity monitoring, and multidimensional outcome assessment, the available evidence remains heterogeneous and does not yet support a single, standardized model of outcome-informed aquatic practice.

The distinction between aquatic competence and participation is particularly relevant. Water competency is an essential outcome because it contributes to safety, autonomy, and access to meaningful aquatic occupations. Kemp et al. identified substantial limitations in water competency among children on the autism spectrum and highlighted the potential influence of sensory characteristics and previous aquatic exposure on participation in swimming and water-related activities [19]. The subsequent AquOTic randomized controlled trial further illustrates the increasing emphasis on structured, occupation-oriented approaches to aquatic intervention aimed at improving water competency [20]. More recently, the same research group demonstrated that individualized occupational therapy-based aquatic interventions can improve caregiver- and therapist-prioritized goals, providing further support for the individualized and outcome-informed approaches that characterize contemporary aquatic practice [21]. However, aquatic competence should not be regarded solely as a performance endpoint. For children and families, the ability to participate safely in swimming, recreation, play, or community water activities may represent the more meaningful outcome. Thus, future aquatic interventions may benefit from explicitly connecting skill acquisition with the occupational contexts in which those skills are intended to be used.

This perspective also challenges the interpretation of sensory responses within aquatic intervention. Sensory characteristics may influence how children respond to immersion, water movement, tactile stimulation, environmental noise, physical contact, or task demands [7,14]. However, the presence of a particular sensory response should not automatically be interpreted as a deficit requiring remediation. Its clinical relevance depends on how that response interacts with the demands of the activity and whether it facilitates or restricts engagement and participation. This distinction is important because several studies have reported changes in sensory [7] or behavioural outcomes [14,34,35] following aquatic intervention, while the mechanisms responsible for these changes remain uncertain. A participation-oriented interpretation therefore requires clinicians to consider sensory responses alongside activity demands, individual preferences, environmental characteristics, and meaningful goals rather than treating sensory characteristics as isolated treatment targets.

The five-phase framework consequently appears most useful as a model of clinical reasoning rather than as a prescriptive intervention sequence. Sensory regulation, aquatic adaptation, skill acquisition, social participation, and broader functional outcomes may interact dynamically rather than occurring in discrete developmental phases. A child may require substantial support for sensory adaptation during one session while simultaneously working on water competency or social participation. Conversely, successful acquisition of aquatic skills may create new opportunities for social interaction and community participation. The framework therefore provides a way of identifying the predominant therapeutic priorities at a particular point in time while retaining sufficient flexibility to accommodate individual developmental trajectories.

### 4.1. Implications for Practice

This narrative review suggests that contemporary aquatic intervention for children with ASD is best understood as a flexible, goal-directed process rather than a sequence of rigid therapeutic phases. The five-phase framework may assist clinicians in identifying the primary priorities guiding intervention while recognizing that children may move between different phases within the same programme or session.

Several practical implications emerge. First, sensory regulation and adaptation to the aquatic environment may provide an important foundation for engagement, particularly when sensory or behavioural characteristics interfere with entering the water, following instructions, tolerating physical contact, or sustaining participation. However, sensory adaptation should be considered in relation to meaningful activity rather than pursued as an isolated therapeutic endpoint.

Second, water safety and aquatic competence should remain central clinical priorities because they directly influence autonomy, risk management, and access to community-based aquatic occupations. The limited water competency reported among children on the autism spectrum reinforces the need for accessible interventions that incorporate individualized motor and sensory adaptations while explicitly addressing water safety.

Third, intervention strategies should be matched to the child’s learning and participation needs. Structured routines, predictable task sequences, visual supports, graded challenges, and individualized instruction may facilitate acquisition of aquatic skills and promote engagement. At the same time, the literature suggests that meaningful aquatic activities can provide opportunities to integrate motor, behavioural, communication, and social goals rather than addressing these domains independently.

Fourth, social outcomes may be particularly relevant when aquatic activities provide naturally occurring opportunities for interaction, cooperation, and shared participation. Studies incorporating peer-, sibling-, group-, or socially oriented aquatic activities suggest that social engagement can be embedded within meaningful aquatic experiences rather than treated exclusively as a separate therapeutic target.

Finally, outcome selection should increasingly reflect the goals that are meaningful to children and families. Motor and aquatic skill measures remain important, particularly for safety and functional competence, but they should ideally be complemented by measures addressing participation, quality of life, sleep, social engagement, behavioural regulation, and other outcomes relevant to everyday life. A multidimensional approach may provide a more clinically meaningful representation of the effects of aquatic intervention than any single outcome domain.

Rather than prescribing a single intervention model, the proposed framework therefore encourages clinicians to select, combine, and adapt strategies according to each child’s strengths, preferences, goals, developmental characteristics, and environmental circumstances. This approach is consistent with the heterogeneous nature of ASD and with the contextual characteristics of aquatic occupations.

### 4.2. Limitations and Future Research

This review should be interpreted in light of several limitations. First, the synthesis is based on heterogeneous studies differing in intervention characteristics, methodological designs, participant profiles, and outcome measures. Many primary studies also provided limited information regarding ASD severity, support needs, cognitive functioning, language abilities, and co-occurring conditions. Consequently, it remains difficult to determine whether specific intervention approaches or outcomes may be more applicable to particular clinical profiles within the heterogeneous autism spectrum. The five-phase framework should be considered a conceptual synthesis of the literature rather than a validated clinical pathway. Second, the available evidence remains uneven across phases, with some domains supported by a larger body of intervention research than others. Third, published studies may provide limited information about the clinical reasoning underpinning intervention selection and adaptation, meaning that important tacit or context-dependent clinical decisions may not be fully represented in the literature.

Future research should therefore move beyond simply evaluating whether aquatic interventions are effective and examine which intervention components are effective, for whom, under which circumstances, and through which mechanisms. Greater consistency in reporting intervention dosage, therapist expertise, environmental conditions, task characteristics, and adaptations would improve reproducibility and facilitate comparison across studies. Intervention studies should also examine whether changes in impairment- or performance-level outcomes translate into meaningful changes in participation and quality of life.

Longitudinal research could further investigate how sensory adaptation, aquatic competence, social engagement, and participation interact over time. Qualitative studies involving clinicians, children, and families may complement quantitative research by examining how goals are established, how intervention priorities change during aquatic programs, and how children and families perceive meaningful change. In addition, physiological and biological measures may provide valuable complementary information, as illustrated by studies examining sleep and inflammatory markers. However, these measures should be integrated within multidimensional models of clinical outcomes rather than treated as independent evidence of therapeutic mechanisms.

## 5. Conclusions

This narrative review provides a conceptual roadmap for understanding the evolution of aquatic intervention research for children with ASD. The five-phase framework highlights a shift from approaches primarily concerned with sensory adaptation and behavioural regulation toward increasingly multidimensional models incorporating aquatic competence, participation, and broader functional outcomes. Importantly, these phases should not be interpreted as a linear sequence but as complementary priorities that may interact according to individual needs and contextual demands.

The synthesis also indicates that the aquatic environment may offer more than a setting for physical exercise. Its distinctive sensory, motor, social, and environmental characteristics create opportunities to address multiple dimensions of functioning and participation. Nevertheless, the current evidence remains heterogeneous, and further research is required to establish how specific intervention components contribute to meaningful outcomes and how changes in individual domains translate into participation.

Ultimately, aquatic clinical reasoning should remain centred on the child’s goals, strengths, preferences, safety, and participation. The proposed framework is intended to support interpretation and clinical decision-making by organizing heterogeneous evidence into a coherent conceptual structure. As such, it may help clinicians link the distinctive properties of the aquatic environment with individualized therapeutic priorities while avoiding a one-size-fits-all approach to intervention.

## Figures and Tables

**Table 1:** Major Clinical Reasoning Paradigms in Aquatic Intervention for children with ASD.

Reasoning Phase	Core Therapeutic Concept	Clinical Reasoning Logic	Primary Outcome Variables	Key Representative References
1. Exploratory and Sensory-Regulatory	Sensory tolerance and water orientation	Can the child tolerate the aquatic medium?	Compliance, water adjustment, affective response	[7]
2. Skill- and Safety-Oriented	Motor competence and safety	Transitioning from aquatic exposure to technical proficiency.	Swimming strokes, rotations, physical fitness	[8,9,10]
3. Structured and Functional	Behavioral control and structured methods	Utilizing water as a structured context supporting behavioral regulation	Postural control, reduction in stereotypies, functional land-based gains	[11,12]
4. Participation- and Social-Oriented	Social affordance and peer interaction	The pool as a mediator for social communication and joint attention.	Social competence scales, peer/sibling interaction, joint attention	[13,14,15,16]
5. Individualized and Outcome-Informed Practice	Neurobiological and multi-system integration	Supporting adaptive functioning and well-being	Executive function, biomarkers (BDNF/Cortisol), sleep architecture	[17,18,19,20,21]

BDNF: brain-derived neurotrophic factor.

**Table 2:** Clinical characteristics and practical application across phases.

Phase	Primary Goals	Delivery & Format	Dose	Results
1. Exploratory and Sensory-Regulatory	Calm alertness; tolerance	1:1 graded immersion; short regulatory loops; minimal demands	1×/week feasible; more if available	Short weekly dosing reduced internalizing/attention and improved state (CBCL)
2. Skill- and Safety-Oriented	Water orientation; discrete safety skills	Buddy + instructor; criterion skill ladders; clear rules	Intensive formats have been associated with faster acquisition of aquatic skills in some studies	5-day program improved swim level; time “out of water” predicts less gain
3. Structured and Functional	Acquisition, generalization, independence	Fixed session arc; visual schedule; repeated skill blocks; fading prompts	1×/week × ≥8 weeks	8-week structured program improved social competence/peers with 1:1 in group
4. Participation- and Social-Oriented	Group participation; turns; roles	1:1 inside group; role-based aquatic games; first–then for regulation	1×/week × ≥8 weeks; boosters	Group tasks increased interaction/engagement; competence gains documented in group formats
5. Individualized and Outcome-Informed Practice	Fidelity; standardized outcomes	Delivered-component checklists; dose logs; pre–post tools	Programmatic blocks; weekly or intensive	Review calls for component reporting/fidelity; program reports detail structured decision-making

**Table 3:** How Aquatic Properties Support Therapeutic Goals Across Phases.

Property	Clinical Function	Example
Buoyancy	Reduced gravitational demands	Motor exploration
Hydrostatic pressure	Sensory regulation	Calm alertness
Viscosity	Resistance and feedback	Motor learning
Warm water	Comfort and participation	Sustained engagement

## Data Availability

Any information supporting the findings of this review is available from the corresponding author upon reasonable request.

## Supplementary Materials

The following supporting information can be downloaded at: https://www.mdpi.com/article/10.3390/children13101327/s1, Table S1. Characteristics of the Studies Included in the Narrative Synthesis and Their Contribution to the Proposed Clinical Reasoning Framework.

## Abbreviations

The following abbreviations are used in this manuscript:

ASD	Autism Spectrum Disorder
SANRA	Scale for the Assessment of Narrative Review Articles
