# Supplementary material for: From Sensory Regulation to Participation: A Clinical Reasoning Framework for Aquatic Therapy in Children with Autism Spectrum Disorder
Source: Children (Basel). 2026 Sep 30;13(10):1327. doi: 10.3390/children13101327 (PMC13702864; doi:10.3390/children13101327)
Supplement: Supplementary file 1 [file children-13-01327-s001.zip › children-4566836-supplementary.pdf]

Supplementary Table S1. Characteristics of the Studies Included in the Narrative Synthesis and Their Contribution to the Proposed Clinical Reasoning Framework.

Study	Year	Design	Participants	Main Focus	Main Outcomes	Paradigm(s)	Dose/Frequency	Key Findings
Killian et al. [7]	1984	Observational	37 children/adolescents with ASD	Water orientation and beginner swim skills	Water adjustment, aquatic orientation	Phase 1	3 weeks	High tolerance and successful progression through water-orientation activities.
Huettig & Darden-Melton [9]	2004	Clinical Report	4 Children with ASD	Adapted swimming instruction	Foundational aquatic skills	Phase 2	NR	Acquisition of foundational aquatic skills following adapted swim instruction.
Yılmaz et al. [8]	2004	Single Case Study	1 child with ASD	Halliwick swimming training	Physical fitness, balance, water orientation	Phase 2–3	10 weeks	Improvements in physical fitness and water orientation.
Yılmaz et al. [22]	2005	Multiple-Probe Design	4 boys with ASD	Aquatic play skills training	Skill acquisition, maintenance, generalization	Phase 2–3	10 weeks; 3×/week	Successful acquisition, maintenance and generalization of aquatic play skills.
Prupas et al. [25]	2006	Program Report	24 Children with ASD and families	Early aquatic intervention	Participation, family engagement	Phase 3	Twice a week/18-week period	Family-oriented aquatic intervention was feasible and promoted participation.
Vonder Hulls et al. [10]	2006	Survey Study	Aquatic clinicians	Perceived benefits of AT	Swim skills, attention, social outcomes	Phase 3	N/A	Clinicians reported perceived benefits in aquatic, behavioural and social domains.

Fragala-Pinkham et al. [28]	2008	Cohort A–B Study	16 children with disabilities including ASD	Group aquatic aerobic exercise	Endurance, swimming participation	Phase 3-4	14 weeks; 2×/week	Improved cardiorespiratory endurance and aquatic participation.
Rogers et al. [24]	2010	Multiple-Probe Design	3 children with ASD/PDD-NOS	Foundational swimming skills	Aquatic skill acquisition	Phase 2	2–3×/week	Foundational swimming skills acquired successfully using constant time delay.
Yılmaz et al. [11]	2010	Multiple-Baseline Design	3 boys with ASD	Halliwick rotation skills	Rotation skills, generalization	Phase 2–3	10 weeks; 3×/week	Halliwick rotation skills acquired and generalized successfully.
Pan [23]	2011	Controlled Cross-Over Study	16 boys with ASD	Water Exercise Swimming Program	Aquatic skills, social behaviour	Phase 2	10 weeks; 2×/week	Improvements in aquatic skills and several social-behavioural outcomes.
Ennis [26]	2011	Pre–Post Study	11 enrolled, 6 completed	PT-directed aquatic intervention	WOTA, QoL, adaptation to water	Phase 3	10 weeks	Improved aquatic competence, water adjustment and quality-of-life indicators.
Fragala-Pinkham et al. [29]	2011	Pilot Study	12 Children with ASD	Group swimming programme	Participation and exercise outcomes	Phase 4	NR	Group aquatic exercise promoted participation and aquatic engagement.
Chu & Pan [30]	2012	Controlled Comparative Study	21 ASD + 21 peers/siblings	Peer- and sibling-assisted aquatics	Interaction behaviours, aquatic skills	Phase 4	16 weeks; 2×/week	Improved interaction behaviours and aquatic skills.

Yanardağ et al. [12]	2013	Multiple-Probe + Pre/Post	3 children with ASD	Video prompting + aquatic exercise	Play skills, motor performance	Phase 3	12 weeks; 3×/week	Video prompting effectively taught aquatic play skills and improved motor performance.
Oriel et al. [31]	2016	ABA Withdrawal Pilot	8 children with ASD	Aquatic exercise programme	Sleep latency, sleep duration	Phase 4	4 weeks; 2×/week	Improvements in sleep latency and sleep duration.
Alaniz et al. [13]	2017	Pilot Intervention	7 children with ASD	Aquatic occupational therapy	Water safety, GAS goals, social skills	Phase 3–4	8–24 h aquatic occupational therapy intervention	Significant gains in water safety skills and individualized aquatic goals.
Caputo et al. [14]	2018	Controlled Intervention	26 children with ASD	Multisystem Aquatic Therapy (CI-MAT)	Vineland, CARS, adaptive functioning	Phase 4-5	10 months; 96 sessions	Improved adaptive functioning, emotional regulation and aquatic skills.
Zanobini & Solari [16]	2019	Controlled Study with Follow-Up	25 children with ASD	Water as Mediator of Communication	Social skills, behavior, aquatic skills	Phase 4	12 sessions; 5 months	Improved relational skills, adaptive behaviours and aquatic abilities.
Mills et al. [34]	2020	Randomized Crossover Pilot	8 children with ASD	Hydrotherapy programme	Mental health, wellbeing, CBCL	Phase 5	4 weeks	Improved mental health-related behavioural outcomes.
Oriel et al. [32]	2020	Mixed-Methods Study	13 Children with ASD	Aquatic social competence intervention	Social competence, participation	Phase 4	NR	Positive changes in social competence and participation.

Ansari et al. [17]	2021	Randomized Controlled Trial	30 children with ASD	Aquatic vs. karate training	Static and dynamic balance	Phase 5	10 weeks; 2×/week	Improved static and dynamic balance.
Sourvinos et al. [15]	2021	Comparative Intervention Study	16 children with ASD	Aquatic Speech and Language Therapy	Receptive/expressive vocabulary	Phase 4	36 sessions; 6 months	Greater gains in receptive and expressive vocabulary than classroom intervention.
Munn et al. [36]	2021	Secondary Program Analysis	86 children with ASD	iCan Swim adapted programme	Swim competency and progression	Phase 5	5 consecutive days; 45–60 min/day	Significant improvements in swim competency levels.
Ansari et al. [33]	2021	Randomized Controlled Trial	40 children with ASD	Aquatic exercise intervention	Sleep quality, IL-1β, TNF-α	Phase 5	10 weeks; 2×/week	Improved sleep quality and reduced inflammatory markers.
Marzouki et al. [35]	2022	Randomized Controlled Trial	22 children with ASD	Technical vs. game-based aquatic training	Motor skills, stereotypy, emotion regulation	Phase 4-5	8 weeks; 2×/week	Improved motor skills and reduced stereotyped behaviour.
Kemp et al. [20]	2024	Cross-Sectional Study	37 children with ASD	Water competency profiling	Water competency, sensory processing	Phase 4-5	Baseline assessment	Lower water competency associated with specific sensory processing profiles.
Kemp et al. [19]	2024	Randomized Controlled Trial	37 children with ASD	AquOTic intervention	Water competency, swim skills	Phase 5	10 weeks	Significant improvements in water competency and swim skills.
Zhao et al. [18]	2024	Randomized Controlled Trial	30 children with ASD	Aquatic exercise intervention	Executive function, BDNF	Phase 4-5	12 weeks; 4×/week; 60–70 min/session	Significant improvements in inhibition control, cognitive flexibility, and

								BDNF levels were observed following aquatic exercise intervention. No significant improvement was found for working memory.
Albadry et al. [27]	2026	Randomized Controlled Trial	50 children with ASD	Aquatic training vs. land exercise	PDMS-2, CARS-2, PedsQL	Phase 3	12 weeks; 3×/week; 45 min/session	Both groups improved, but aquatic training produced greater improvements in gross motor function, quality of life, and autism-related outcomes than land-based exercise.
Kemp et al. [21]	2026	Cohort Embedded in RCT	37 children with ASD	AquOTic occupational therapy	COPM, GAS, individualized goals	Phase 5	10 weeks; 1×/week; 60 min/session	Significant improvements were observed in caregiver- and therapist-defined individualized water competency goals, with clinically meaningful improvements in COPM and GAS outcomes following intervention.

ASD, Autism Spectrum Disorder; AT, Aquatic Therapy; AquOTic, Occupational Therapy–Based Aquatic Intervention Program; BDNF, Brain-Derived Neurotrophic Factor; CBCL, Child Behavior Checklist; CARS, Childhood Autism Rating Scale; CI-MAT, Multisystem Aquatic Therapy (Caputo–Ippolito Method); COPM, Canadian Occupational Performance Measure; GAS, Goal Attainment Scaling; IL-1β, Interleukin-1 Beta; PDMS-2, Peabody Developmental Motor Scales, Second Edition; PDD-NOS, Pervasive Developmental Disorder–Not Otherwise Specified; PedsQL, Pediatric Quality of Life Inventory; PT, Physical Therapy; QoL, Quality of Life; TNF-α, Tumor Necrosis Factor Alpha; WOTA, Water Orientation Test–Alyn; N/A, Not applicable; NR, not reported.
